# Incubation of Negative Affect during Protracted Alcohol Withdrawal Is Age-, but Not Sex-Selective

**DOI:** 10.3390/brainsci10060405

**Published:** 2020-06-26

**Authors:** C. Leonardo Jimenez Chavez, Michal A. Coelho, Lindsey W. Brewin, Isaiah Swauncy, Tori Tran, Taylor Albanese, Angie Laguna, Ivette Gabriela, Karen K. Szumlinski

**Affiliations:** 1 Department of Psychological and Brain Sciences, University of California Santa Barbara, Santa Barbara, CA 93196-9660, USA; clj@ucsb.edu (C.L.J.C.); michalcoelho@gmail.com (M.A.C.); lindseybrewin@ucsb.edu (L.W.B.); Isaiah_swauncy@yahoo.com (I.S.); toritran@gmail.com (T.T.); talbanese@ucsb.edu (T.A.); angielaguna@ucsb.edu (A.L.); 2 Department of Psychology, California State University Dominguez Hills, Carson, CA 90747-0001, USA; ivettgabriella@gmail.com; 3 Department of Molecular, Cellular and Developmental Biology and the Neuroscience Research Institute, University of California Santa Barbara, Santa Barbara, CA 93106-6050, USA

**Keywords:** binge-drinking, negative affect, anxiety, sex differences, adolescence, withdrawal

## Abstract

A prior history of excessive drinking induces a negative affective state in both humans and laboratory rodents, the manifestation of which varies with the age of drinking-onset. In adolescent male mice, negative affect incubates over the course of a 30-day alcohol withdrawal period. In contrast, the negative affect exhibited by adult male mice is robust at 1 day withdrawal, but dissipates with the passage of time. As females tend to consume more alcohol than males, we aimed to explore the affective disturbances exhibited by adolescent and adult C57BL/6J mice of both sexes during more protracted alcohol withdrawal and to relate any behavioral changes observed to plasma corticosterone levels as a biochemical index of stress. Male and female, adolescent and adult, mice were subjected to 14 consecutive days of binge alcohol-drinking using a multi-bottle-choice Drinking-in-the-Dark (DID) procedure (5, 10, 20 and 40% *v*/*v*). Age- and sex-matched control mice consumed water only. On either withdrawal day 1 or 70, subgroups of animals were subjected a to 1-day behavioral test battery that included the light–dark box shuttle test, marble-burying test, and Porsolt forced swim test. As expected, adolescent mice consumed more alcohol than adults and females consumed more alcohol than males. However, despite binge-like levels of alcohol consumption, we detected relatively few signs of alcohol withdrawal-induced negative affect and there was no correlation between affective behavior and circulating corticosterone levels. We discuss these findings within the context of our published work, highlighting procedural differences that might account for the relatively weak effect of binge-drinking history upon anxiety and depressive-like behavior observed herein.

## 1. Introduction

Binge-drinking is the most common form of alcohol abuse amongst adolescents. The National Institute of Alcohol Abuse and Alcoholism (NIAAA) cites that 90% of all underage drinkers within the United States have engaged in binge-drinking behaviors [1], with an estimated 1 million adolescents engaging in frequent binge-drinking episodes [2]. Binge-drinking is a pattern of high alcohol consumption that results in blood alcohol concentrations (BAC) ≥ 0.08 g/dL, in an approximately two-hour period [1]. For humans, this usually occurs after 4 drinks for adult women and 5 drinks for adult men [1]. The prevalence of binge-drinking in adolescents is concerning as adolescence is a critical period of brain development that occurs in between the ages of 12–17 in humans and approximately postnatal days (PND) 28–50 in laboratory rodents. During this period, the brain undergoes robust structural and functional changes, including alterations in neuronal connectivity and synaptic plasticity [3]. With these neuroadaptations come changes in behavior, including increased risk-taking, impulsivity, and vulnerability to stressors [4,5,6,7]. Coupled with environmental and social influences, these adolescent-related behavioral phenotypes have been theorized to increase drug abuse propensity, including excessive alcohol-drinking [8]. This increased propensity to consume alcohol is augmented by the fact that adolescents tend to be significantly less sensitive to alcohol’s negative reinforcing properties than adults, including “hang-over” and increased negative affect during early alcohol withdrawal [6,9,10].

Excessive alcohol experience during adolescence impinges upon neurodevelopment, particularly that within the mesocorticolimbic system regulating motivation, emotion, learning, and memory, as well as volitional control over behavior [11,12]. Consequently, excessive alcohol consumption during adolescence is associated with decreased academic performance [13,14], increased criminal activity [15], increased vulnerability to develop affective and substance use disorders in later life, including Alcohol Use Disorder (AUD) [6,16,17,18,19]. Additionally, individuals with an early age of drinking-onset suffer from alterations in hypothalamo–pituitary–adrenal (HPA) axis function, which also contributes to the manifestation of affective disorders in humans [20,21]. Although it is difficult to dissect a causal link between early binge-drinking history and the manifestation of mental disorders in later life, support for a direct cause–effect relationship can be derived from the animal literature [22,23]. As an example from our own laboratory, adult, male mice with a prior history of binge-drinking during adolescence exhibit both a hyper-anxious phenotype and augmented alcohol intake, relative to both water-drinking controls and mice with a more recent binge-drinking history during adulthood [24]. Further, in so far as we have investigated in male mice, the age-related differences in the temporal manifestation of withdrawal-induced negative affect reflect an interaction between the age of binge-drinking onset and time-dependent changes in the expression and function of glutamate receptor-related proteins within extended amygdala structures [10,24,25,26]. Such data argue that, at least in male mice, adolescent-onset binge-drinking negatively impacts the development of the mesocorticolimbic glutamate system within the major neurocircuits gating emotion and motivation.

Globally, a sex difference exists with respect to the prevalence of affective disorders, with females being twice as likely as males to be diagnosed with an anxiety-related disorder, irrespective of past or concurrent drug abuse [27]. The results of the extant literature focused on how biological sex and/or gender interacts with the age of drinking-onset to influence binge-drinking propensity/the development of AUD are less consistent, with data from the human literature pointing to sociocultural factors as major influences on study outcomes [28,29,30]. In contrast, robust sex differences exist with respect to alcohol consumption in laboratory animals (including binge-drinking), with female non-human primates, rats, and mice consuming more alcohol than age-matched males in the majority of studies [30,31,32]. Female laboratory rodents also escalate alcohol-taking more quickly and exhibit greater relapse-like drinking than their male counterparts [30,31,32]. While fewer in number, some animal studies have examined the interactions between biological sex and age of drinking-onset with respect to binge-drinking-related outcomes [32,33,34,35,36]. Of direct relevance to this project, we demonstrated recently that akin to adult males [10], adult female B6 mice with a 2-week history of binge-drinking exhibit robust sings of negative affect during early (1 day) withdrawal [35]. However, in contrast to males [11,25], this negative affective state persists in adult females for at least 30 days post-drinking [35]. We also detected what might be a sex difference in the onset of a negative affective state during withdrawal in binge-drinking adolescents; female mice with a prior history of adolescent binge-drinking exhibit signs of a negative affective state in both early and later withdrawal [35], while that observed in males incubates with the passage of time post-drinking [24]. Taken together, these findings regarding the interaction between sex and age of binge-drinking onset suggest that females may be more susceptible than males to developing a long-lasting change in emotionality during protracted alcohol withdrawal that warranted a direct investigation.

Herein, we examined sex differences in the effects of withdrawal from a 2-week binge-drinking history either during adolescence or adulthood upon anxiety- and depression-like behavior in B6 mice. As in our published work [10,24,35], a subset of mice was tested for anxiety- and depression-like behavior at one day post-drinking to test the hypothesis that adolescent female “bingers” will exhibit an earlier onset of negative affective signs, than their male binge-drinking counterparts. To extend the results of our prior studies using a 30-day withdrawal period [10,24,35], another subset of mice was tested for affective behavior at 70 days post-drinking. This was done to probe (1) the permanency of the effects of adolescent binge-drinking upon emotionality and (2) potential sex differences in the longevity of the binge-drinking effect in adult animals. Baseline and test-induced increases in plasma corticosterone (CORT) were examined to relate behavioral differences to the function of the hypothalamo–pituitary–adrenal (HPA) axis, based on evidence that sex- and age-related differences exist with respect to HPA function that may have relevance for manifestation of binge-drinking, the etiology of AUD and affective disorder comorbidity [32,36,37,38,39,40].

## 2. Materials and Methods

### 2.1. Subjects

This study employed a combination of male and female, adolescent (postnatal day PND = 28/29) and adult (PND = 56) C57BL/6J (B6) mice obtained from The Jackson Laboratory (Sacramento, CA, United States) or bred in-house in the Psychology vivarium at the University of California, Santa Barbara (UCSB) from breeder pairs originally obtained from the Jackson Laboratory. Mouse origin was based on practical considerations at the time of testing of the final cohorts of this large-scale study (fecundity of breeders, number of age-appropriate offspring available for testing) and was not considered to be a major factor in determining binge-drinking behavior, based off prior work conducted by our laboratory [35]. At PND 21, mice bred in-house were weaned from their litters and placed with different littermates of the same age and sex in groups of 4 in polycarbonate cages. As in our prior work [10,24,35], commercially sourced mice arrived at 21–22 days of age and were housed in same-age and -sex groups of 4. Mice were allowed 7 days to acclimate to a colony room, where they were housed under a reverse 12 h-light/dark cycle (lights off at 10:00 h) in a climate and humidity-controlled vivarium. The animals were identified using a combination of tail and ear markings and the mice from the two different sources were assigned to the different conditions as equally as possible. Food and water were available ad libitum to all the animals except during the 2-h alcohol-drinking period. All the cages were lined with sawdust bedding and nesting materials in accordance with vivarium protocols. All routine cage cleaning/bedding changes were suspended at least 5 days prior to testing for behavioral signs of negative affect to eliminate potential confounds due to the change in the home-cage environment and foreign handling by the vivarium staff. All experimental procedures were in compliance with The Guide for the Care and Use of Laboratory Animals (2014) and approved by the Institutional Animal Care and Use Committee of the University of California, Santa Barbara.

### 2.2. Drinking-In-The-Dark (DID) Procedures

Approximately half of the mice (*N* = 102) were subjected to 14 consecutive days of binge- drinking using a multi-bottle-choice DID procedure that involved concurrent access to unsweetened ethanol at 5, 10, 20, and 40% (*v*/*v*) concentrations [10,24,35]. On each drinking day, all animals were transferred into a dark, non-colony, procedural room within the vivarium, and the mice slated to binge-drink were transferred from their home cages to an individual drinking cage lined with sawdust bedding, situated on a free-standing rack and allowed to acclimate to the drinking cage for 1 h before bottle presentation [35]. After cage habituation, sipper tubes containing the alcohol solutions were placed on the drinking cage with the location of sipper tubes randomized daily and animals were allowed 2-h access (14:00–16:00 h). At 16:00 h, the sipper tubes were removed from the drinking cages and the binge-drinking mice were then transferred back into their home cages. To facilitate study throughput, water control mice remained in their home cages, but were placed onto the same free-standing rack as the binge-drinking mice for the 3-h period. The water-drinking controls were not singly housed in this study, based on the collection of results from prior work, admittedly conducted exclusively in male mice, indicating comparable anxiety/depression-like behavior between water controls singly-housed for 3 h/day during the drinking procedures versus those merely transferred to the free-standing rack during this period [10,24,25,26,41,42,43]. At the end of each drinking session, all the mice were returned the colony room. Mice were weighted every 3–4 days during the drinking procedures and their recorded weight was used in to calculate alcohol intake.

### 2.3. Blood Alcohol Concentration Sampling

**Figure 1:**
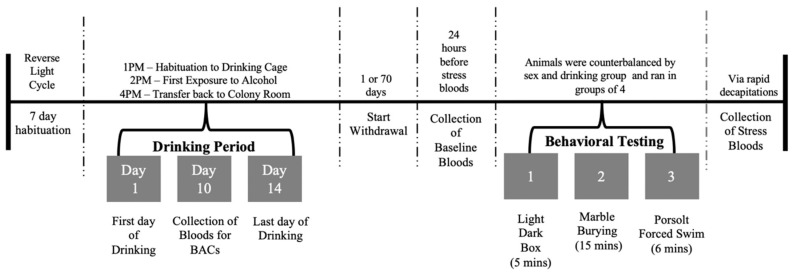
Summary of the procedural time-line for this study.

Immediately after the 2-h alcohol-drinking period on the 10th drinking day, submandibular blood samples were collected from alcohol-drinking mice only. This sampling time-point was selected as the alcohol intake had stabilized, thereby providing a more accurate measure of their average daily alcohol consumption. Additionally, this time-point allowed sufficient recovery time prior to behavioral testing at the 1-day withdrawal time-point. A time-line of all the procedures employed in this study is provided in Figure 1 below.

### 2.4. Gas Chromatography

Headspace gas chromatography is the gold standard for ethanol analysis due to its effectiveness and accuracy in determining levels in various substances, including blood [44]. BACs were determined using a Shimadzu GC-2014 gas chromatography system (Shimadzu, Columbia, MD, USA) and the data was determined via the GC Solutions version 2.10.00 software. Samples were diluted at 1:9 with non-bacteriostatic saline (50 μL of sample). Acetone and dichloromethane were used as the pre-solvents due to their lower boiling point versus ethanol. Each sample was tested within 1-week of blood collection to reduce the potential for ethanol evaporation during storage. The determination of ethanol from each sample was derived using the standard curve equation determined prior to analyses of the samples. A new standard curve was formulated for each cohort of blood samples to ensure maximal accuracy. After the ethanol peak area was determined, the peak area was used to determine the ethanol concentration and subsequently the percent of ethanol in the blood. The BACs were then correlated with the alcohol intake observed on day 10 of drinking, which is when the blood was sampled.

### 2.5. Baseline and Stressor-Induced CORT Assay

To assay circulating plasma CORT levels, submandibular blood samples (50 μL) were collected from all the mice 24 h before behavioral testing for negative affect (for baseline CORT) at approximately 10:00 h (±30 min) and trunk blood was collected immediately following behavioral testing at approximately 17:00 h (±30 min) to index stressor-induced changes in circulating CORT. For all the animals tested at 1-day withdrawal, the baseline blood samples were collected before mice were habituated to the drinking cages on the final (14th) day of drinking. All the bloods samples were collected in a blood collection tubes lined with lithium heparin (BD Vacutainer, Mississauga, ON, Canada) and centrifuged at 10,500 rpm at 4 °C for 20-min to obtain plasma. The extracted plasma sample was kept frozen at −80 °C until assayed. Duplicate samples were analyzed using the DetectX Corticosterone Immunoassay kit K014-H5 (Arbor Assays, Ann Arbor, MI, USA). CORT levels were determined following the manufacturer’s recommended instructions.

### 2.6. Behavioral Testing

In prior work, male B6 mice with a history of adolescent-onset binge-drinking exhibit no signs of negative affect when tested at one day withdrawal but exhibit robust anxiety- and depressive-like behaviors when tested at 30 days withdrawal [10,24]. In contrast, male B6 mice with a history of adult-onset binge-drinking exhibit signs of hyper-anxiety at 1-day withdrawal, but this negative affective state is no longer detectable at 30 days withdrawal [10,24]. Female B6 mice with a history of either adolescent- or adult-onset binge-drinking exhibit signs of anxiety- and depressive-like behaviors during both early and later withdrawal [35]. To directly examine for sex by age interactions in the effects of binge-drinking upon negative affect during alcohol withdrawal, we conducted a 1-day behavioral test battery consisting of the light–dark shuttle-box test, the marble burying test, and the Porsolt forced swim test. We and others have shown that these behavioral assays are sensitive to withdrawal-induced changes in negative affect, as well as age-related differences therein [10,24,35,45,46].

#### 2.6.1. Light–Dark Shuttle-Box

The light–dark shuttle-box was used to measure anxiety-like behaviors, with decreased activity in the light-side interpreted as reflecting an anxiety-like phenotype [47,48]. Animals were placed into a polycarbonate box measuring 46 cm in length × 22 cm in width × 24 cm in height. The box was divided into two environments, one side is white with no lid and the other side was black with a black lid (respectively, light versus dark side). During the experiment, the two environments were accessible through a central divider with an opening. The animals were first introduced to the dark environment by the experimenter and using AnyMaze^TM^ tracking software (Stoelting Co., Wood Dale, IL, USA), our dependent measures of latency to enter the light side, total time spent in the light side and total number of light entries were recorded over a 5-min period. The boxes were cleaned in-between each use with Rescue Disinfectant Veterinary Wipes (Virox Animal Health, Oakville, ON, Canada). Immediately upon completion of this assay, the animals were transferred back into their home cages and transported to a distinct procedural room for marble-burying testing.

#### 2.6.2. Marble-Burying Test

The marble-burying test is particularly sensitive to the effects of alcohol withdrawal based on our prior work [10,24,25,26,35,41,42,43]. Mice were placed in polycarbonate cage (12 cm × 8 cm × 6 cm), with 5-cm deep sawdust bedding on top of which marbles were arranged equidistantly. The percent of marbles buried (i.e., 75% covered by bedding) was determined after a 15-min session. The behavior of the animals was also video-recorded using AnyMaze^TM^ tracking software and the total time spent burying and the latency to start burying was recorded by a blind observer using a stopwatch. At the end of the marble-burying session, the animals were returned to their home cages and transported to a 3rd procedural room for forced swim testing.

#### 2.6.3. Porsolt Forced Swim Test

The Porsolt Forced Swim test is commonly employed assay for depressive-like behaviors and their reversal by anti-depressant treatments [49]. Excessive swimming behavior in this assay can be reversed by pretreatment with anxiolytic medications and thus, has been used by our group as an additional measure of anxiety-like behavior [26,33,34]. In our paradigm, an 11-cm diameter cylindrical glass container is filled with room temperature water and animals are tested over a 6-min period during which AnyMaze^TM^ tracking software determined the latency to first immobile episode, total time spent immobile, and the number of immobile episodes. Immobility is defined as the lack of vertical or horizontal displacement of the animal’s center of gravity for at least 5-s. Upon the conclusion of this assay, animals were euthanized by rapid decapitation and trunk blood collected to index stressor-induced increases in plasma CORT.

### 2.7. Statistical Analysis

All statistical tests were conducted using the IBM SPSS Statistics software (IBM Corp. IBM SPSS Statistics for Macintosh, Version 24.0. Armonk, NY, USA). All graphs were created using the GraphPad Prism software (GraphPad Prism Software for Macintosh, Version 8.01. La Jolla, CA, USA). Previous findings from our laboratory indicate an age-dependent effect of alcohol withdrawal upon anxiety [10,24,35]. Thus, to increase the statistical power to detect lower level sex differences, the data for the Age and Withdrawal factors were analyzed separately using between-subjects univariate Analyses of Variance (ANOVAs). For all Sex × Drinking ANOVAs conducted on the data for affective behavior, α = 0.05 was used and post-hoc *t*-test comparisons were employed when appropriate. Based on our prior evidence for age-related differences in the effects of binge-drinking upon anxiety-related behavior of male mice, we classified all behavioral ANOVAs rendering an α = 0.05 − 0.10 as a statistical and notable trend [10,24,25,26,42,43]. To ensure that the mice tested in early versus later withdrawal exhibited comparable alcohol intake, the average total alcohol intake over the 14-day drinking period was analyzed using an Age × Sex × Withdrawal ANOVA, with α = 0.05. The CORT data were also analyzed separately for adolescent and adult mice using a Sex × Drinking × Withdrawal ANOVA with α = 0.05. We normalized the stressor-induced changes in CORT to the baseline CORT levels for each subject and conducted a similar univariate analysis. Pearson’s correlational tests were conducted to correlate: (1) BACs with alcohol intake; (2) alcohol intake with our CORT and behavioral measures; and (3) our CORT measures with behavior. As no a priori hypothesis regarding sex differences was established, α = 0.05 was used for all CORT- and BAC-related analyses.

## 3. Results

### 3.1. Alcohol Intake and Blood Alcohol Concentrations

**Figure 2:**
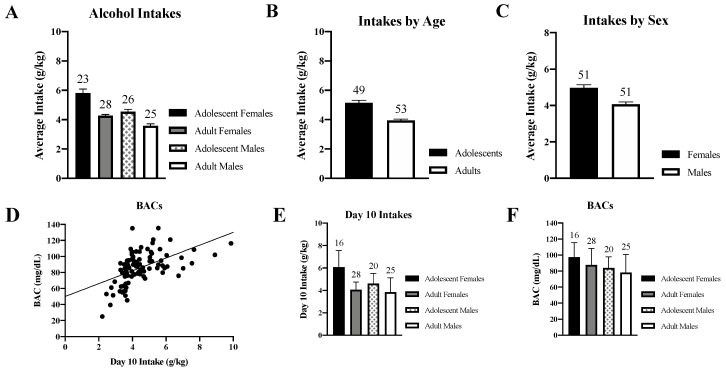
Summary of group differences in alcohol intake under our Drinking-in-the-Dark (DID) procedures. (**A**) Comparison of the average alcohol intake (g/kg) of the adult and adolescent male and female mice over the course of the 14-day drinking period. (**B**) Data from 1a, collapsed across Sex to illustrate the main Age effect detected by ANOVA. (**C**) Data from 1a, collapsed across Age to illustrate the main Sex effect detected by ANOVA. (**D**) Correlation between alcohol intake and BACs collected on Day 10 of drinking. (**E**) Comparison of group differences in alcohol intake on Day 10 of drinking when blood was sampled. (**F**) Comparison of group differences in the average BACs attained on Day 10 of drinking. The data represent the means ± SEMs for the numbers of mice indicated in each panel.

Binge-drinking animals consumed on average 4.52 ± 1.1 g/kg alcohol during the 14-day drinking period. The univariate Age × Sex × Withdrawal ANOVA resulted in no significant interactions (*p*’s > 0.07; Figure 2A). However, we detected a main Age effect (*F*(1,94) = 54.48, *p* < 0.001), which reflected more alcohol consumption in adolescent versus adult mice (Figure 2B) and a main Sex effect (*F*(1,94) = 34.33, *p* < 0.001) that reflected more alcohol consumption in females versus males (Figure 2C). When all the animals were considered, a Pearson’s correlation showed a positive relationship between BAC levels and alcohol intake (*r* = 0.529, *p* < 0.0001, Figure 2D), with a pattern of group differences in line with those observed for the total alcohol intake (Figure 2E vs. Figure 2A).

### 3.2. Light–Dark Shuttle-Box

#### 3.2.1. Latency to Enter the Light Side

**Figure 3:**
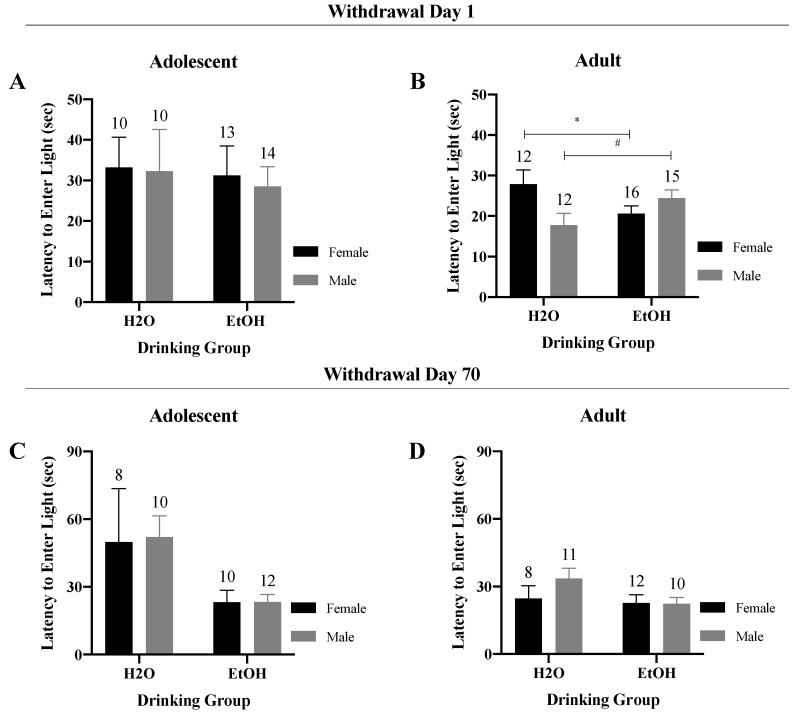
Summary of the results of the Sex × Drinking ANOVA conducted on the data for the latency to enter the light-side of the light–dark shuttle-box. (**A**) Depiction of the results for the adolescent-onset mice at 1-day withdrawal. (**B**) Depiction of the Sex × Drinking interaction observed for the adult-onset mice at 1-day withdrawal. (**C**) Depiction of the results for the adolescent-onset mice at 70-days withdrawal. (**D**) Depiction of the results for the adult-onset mice at 70-days withdrawal. The data represent the means ± SEMs for the numbers of mice indicated in each panel. * *p* < 0.05 for female H2O vs. female EtOH; # *p* < 0.05 for male H2) vs. male EtOH.

A Sex × Drinking ANOVA conducted on the latency to enter the light side of the shuttle-box yielded no significant interaction for the adolescent mice in early withdrawal (Figure 3A) (*F*(1,43) = 0.01, *p* = 0.91). However, a significant Sex × Drinking interaction was observed for the adult-onset animals tested in early withdrawal (Figure 3B) (*F*(1,51) = 7.53, *p* = 0.01). Deconstruction of this interaction did not reveal any significant group differences; however, a statistical trend in the latency to enter the light side was observed for female mice in that female binge-drinkers exhibited a shorter latency to enter the light side, compared with their water-drinking counterparts (*t*(26) = 1.97, *p* = 0.06). In contrast, male binge-drinkers exhibited a longer latency to enter the light side than their water-drinking counterparts (*t* (25) = 1.92, *p* = 0.07).

For adolescent mice in protracted withdrawal, no significant Sex × Drinking interactions were observed (Figure 3C) (*F*(1,36) = 0.01, *p* = 0.93). However, a significant main effect of drinking was noted for these animals (*F*(1,36) = 6.29, *p* = 0.02), reflecting a shorter latency to enter the light side for the adolescent binge- versus water-drinking animals. No significant Sex x Drinking interaction or main effects were confirmed for the adult mice in protracted withdrawal (Figure 3D) (*F*(1,37) = 1.23, *p* = 0.27).

#### 3.2.2. Time Spent in the Light Side

**Figure 4:**
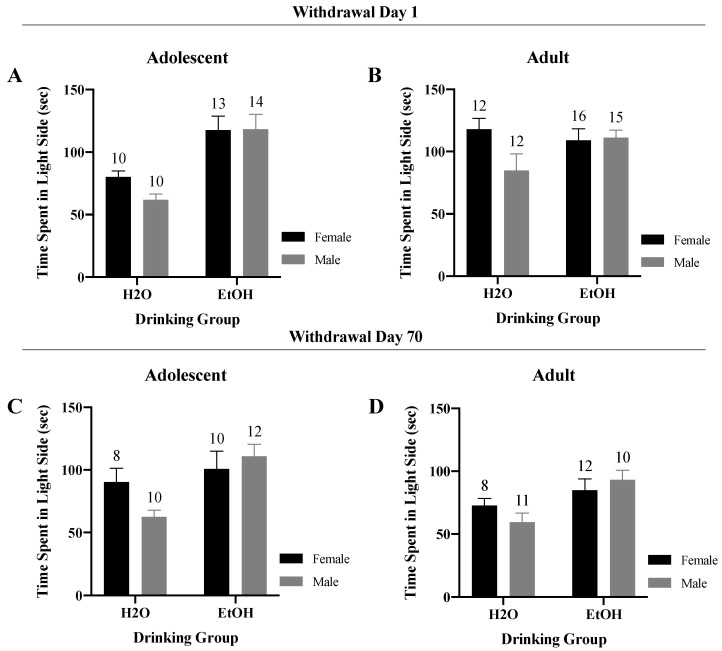
Summary of the results for the Sex × Drinking ANOVA observed for the total time spent in the light-side in the light–dark shuttle-box test. (**A**) Depiction of the results for the adolescent-onset mice at 1-day withdrawal. (**B**) Depiction of the results observed for the adult-onset mice at 1-day withdrawal. (**C**) Depiction of the results for the adolescent-onset mice at 70-days withdrawal. (**D**) Depiction of the results for the adult-onset mice at 70-days withdrawal. The data represent the means ± SEMs for the numbers of mice indicated in each panel.

A Sex x Drinking ANOVA indicated no significant interaction for the total time spent in the light side of the shuttle-box by the adolescent-onset mice tested in early withdrawal (Figure 4A) (*F*(1,43) = 0.93, *p* = 0.34). However, a significant main effect of Drinking was observed for these animals (*F*(1,43) = 22.54, *p* < 0.001), which reflected more time spent in the light-side by the binge-drinkers versus the water controls. For the adult-onset mice tested in early withdrawal, the interaction exhibited a statistical trend (Figure 4B) (*F*(1,51) = 3.50, *p* = 0.07), which reflected more time spent in the light-side by alcohol- versus water-drinking males (*t* (25) = 1.95, *p* = 0.06). In contrast, no significant alcohol-related trend was observed for female mice tested in early withdrawal (*t*-test: *p* = 0.51).

Analyses of the total time spent in the light side for adolescent mice tested in protracted withdrawal also revealed a Sex x Drinking trend (Figure 4C) (*F*(1,36) = 3.17, *p* = 0.08). In adolescent males, binge-drinkers spent significantly more time in the light-side compared to their water-drinking controls (*t* (20) = 4.13, *p* = 0.001). In contrast, no effect of Drinking was observed for the adolescent females tested in protracted withdrawal (*t*-test, *p* = 0.58). No significant two-way interaction or trend was observed for the adult-onset mice tested in protracted withdrawal (Figure 4D) (*F*(1,37) = 1.84, *p* = 0.18). However a significant main effect of Drinking was detected in the adult animals (*F*(1,37) = 8.44, *p* = 0.01), with adult binge-drinkers spending more time in the light side versus the adult water controls.

#### 3.2.3. Number of Light Entries

**Figure 5:**
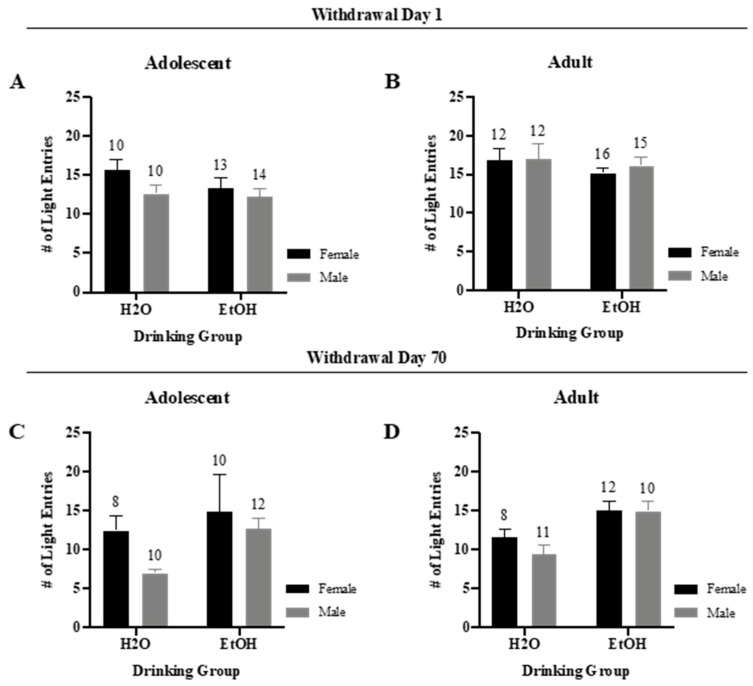
Summary of the results for the Sex × Drinking ANOVA observed for the total number of entries to the light-side in the light–dark shuttle-box test. (**A**) Depiction of the results for the adolescent-onset mice at 1-day withdrawal. (**B**) Depiction of the results observed for the adult-onset mice at 1-day withdrawal. (**C**) Depiction of the results for the adolescent-onset mice at 70-days withdrawal. (**D**) Depiction of the results for the adult-onset mice at 70-days withdrawal. The data represent the means ± SEMs for the numbers of mice indicated in each panel.

Examination of the total number of light entries in early withdrawal indicated no significant interaction or trends for either the adolescent-onset (Figure 5A) (*F*(1,43) = 0.76, *p* = 0.39) or the adult-onset mice (Figure 5B) (*F*(1,51) = 0.09, *p* = 0.76). However, a trend for a main effect of Sex was observed for the adolescent mice tested in early withdrawal (*F*(1,43) = 2.98, *p* = 0.09), that reflected more light entries in female versus male mice.

For the animals in protracted withdrawal, no significant interaction or trend was observed for the adolescent mice (Figure 5C) (*F*(1,36) = 0.38, *p* = 0.54) or for the adult mice (Figure 5D) (*F*(1,37) = 0.80, *p* = 0.38); however a significant main effect of Drinking was noted for the adult-onset mice (*F*(1,37) = 13.65, *p* = 0.001) indicating that the binge-drinkers had a higher number of light entries than water-controls.

### 3.3. Marble Burying Test

#### 3.3.1. Latency to Start Burying

**Figure 6:**
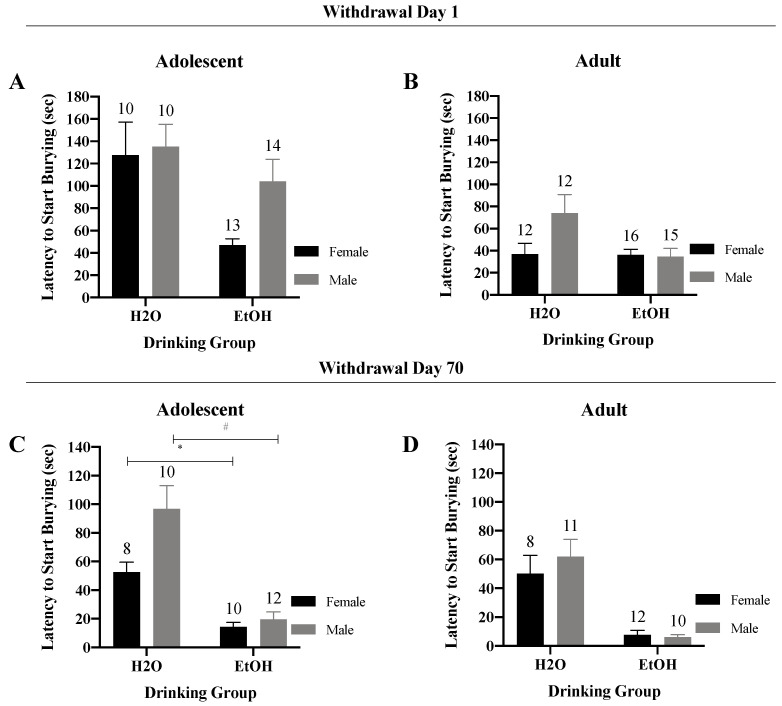
Summary of the results for the Sex × Drinking ANOVA observed for the latency to start burying marbles in the marble-burying test. (**A**) Depiction of the results for the adolescent-onset mice at 1-day withdrawal. (**B**) Depiction of the results observed for the adult-onset mice at 1-day withdrawal. (**C**) Depiction of the Sex × Drinking interaction for the adolescent-onset mice at 70-days withdrawal. (**D**) Depiction of the results for the adult-onset mice at 70-days withdrawal. The data represent the means ± SEMs for the numbers of mice indicated in each panel. * *p* < 0.05 for female H2O vs. female EtOH; # *p* < 0.05 for male H2) vs. male EtOH.

Examination of the latency to start burying for adolescent-onset mice in early withdrawal indicated no significant Sex × Drinking interaction (Figure 6A) (*F*(1,43) = 1.58, *p* = 0.22). However, there was a significant main effect of Drinking (*F*(1,43) = 8.11, *p* = 0.01), which reflected a shorter latency to start burying in alcohol- versus water-drinking adolescents. For the adult-onset mice, the results of the two-way ANOVA indicated a statistical trend for the interaction (Figure 6B) (*F*(1,51) = 3.76, *p* = 0.06), which reflected a shorter latency to bury in alcohol- versus water-drinking males tested in early withdrawal (*t* (25) = 2.30, *p* = 0.03), but no alcohol-related difference in adult females. (*p* = 0.92).

The Sex x Drinking ANOVA for the adolescent mice tested in protracted withdrawal indicated a significant interaction (Figure 6C) (*F*(1,36) = 4.38, *p* = 0.04). Deconstruction of this interaction along the Sex factor revealed a shorter latency to bury in alcohol- versus water-drinking females (*t* (16) = 5.29, *p* < 0.001) and males (*t* (20) = 4.90, *p* < 0.001). A two-way interaction was not observed for the adult-onset mice tested in protracted withdrawal (Figure 6D) (*F*(1,37) = 0.65, *p* = 0.43). However, a significant main effect of Drinking Group was apparent that reflected a shorter bury latency in binge-drinking mice versus water-drinking counterparts (*F*(1,37) = 34.76, *p* < 0.001).

#### 3.3.2. Time Spent-Burying

**Figure 7:**
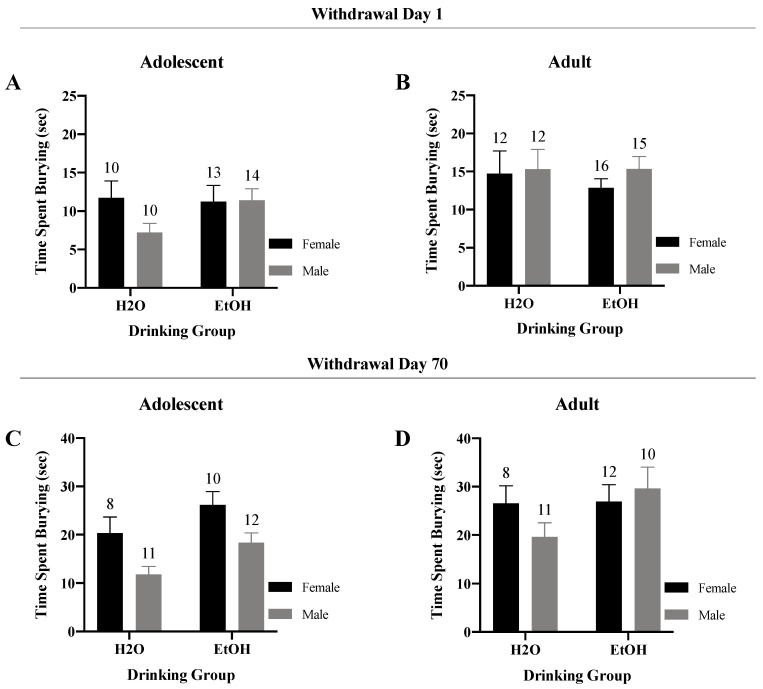
Summary of the results for the Sex × Drinking ANOVA observed for the total time spent burying marbles in the marble-burying test. (**A**) Depiction of the results for the adolescent-onset mice at 1-day withdrawal. (**B**) Depiction of the results observed for the adult-onset mice at 1-day withdrawal. (**C**) Depiction of the results for the adolescent-onset mice at 70-days withdrawal. (**D**) Depiction of the results for the adult-onset mice at 70-days withdrawal. The data represent the means ± SEMs for the numbers of mice indicated in each panel.

A Sex × Drinking ANOVA conducted on the time-spent burying marbles by adolescent-onset mice tested in early withdrawal indicated no significant interactions or noteworthy trends (Figure 7A) (*F*(1,43) = 1.64, *p* = 0.21). Similarly, the results of the two-way ANOVA for the adult-onset mice in early withdrawal uncovered no significant interactions or trends were detected for the adult-onset mice tested in early withdrawal (Figure 7B) (*F*(1,51) = 0.20, *p* = 0.65). No observable main effects were found for either age group for this variable (*p*’s > 0.05).

In protracted withdrawal., analyses of the data for adolescent-onset mice revealed a significant main effect of both Sex (*F*(1,36) = 11.27, *p* = 0.002) and Drinking Group (*F*(1,36) = 6.43, *p* = 0.02), but no significant Sex × Drinking interaction (Figure 7C) (*F*(1,36) = 0.02, *p* = 0.88). Female adolescent-onset mice spent more time burying in protracted withdrawal than males, with binge-drinking mice spending more time burying than water controls (Figure 7C). Analyses of the data for adult-onset mice did not yield a significant Sex × Drinking interaction nor any other notable trends for this variable (Figure 7D) (*F*(1,37) = 1.72, *p* = 0.20).

#### 3.3.3. Percent of Marbles Buried

**Figure 8:**
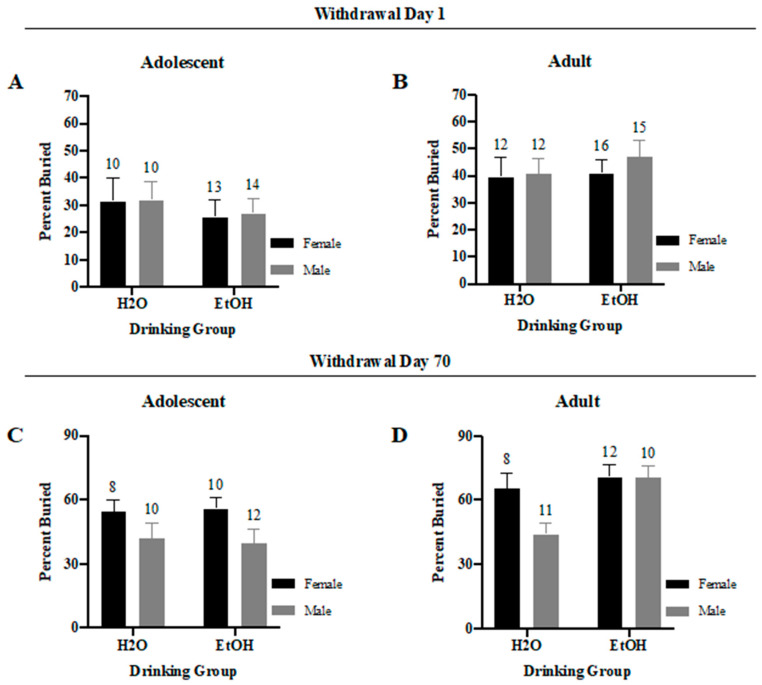
Summary of the results for the Sex × Drinking ANOVA observed for the percent of marbles buried. in the marble-burying test. (**A**) Depiction of the results for the adolescent-onset mice at 1-day withdrawal. (**B**) Depiction of the results observed for the adult-onset mice at 1-day withdrawal. (**C**) Depiction of the results for the adolescent-onset mice at 70-days withdrawal. (**D**) Depiction of the results for the adult-onset mice at 70-days withdrawal. The data represent the means ± SEMs for the numbers of mice indicated in each panel.

A Sex × Drinking ANOVA indicated no significant interactions for either the adolescent-onset (Figure 8A) (*F*(1,43) = 0.004, *p* = 0.95) or adult-onset (Figure 8B) (*F*(1,51) = 0.16, *p* = 0.69) mice in early withdrawal. Similarly, no significant interaction was detected for the adolescent-onset mice in later withdrawal (Figure 8C) (*F*(1,36) = 0.10, *p* = 0.75); although there was a significant main effect of Sex (*F*(1,36) = 5.10, *p* = 0.03), which reflected a higher percentage of marble buried in the adolescent females versus males tested during protracted withdrawal. Results for the adult-onset mice tested in later withdrawal yielded a statistical trend for a Sex × Drinking interaction (Figure 8D) (*F*(1,37) = 3.44, *p* = 0.07). This trend reflected more marble-burying in male binge-drinkers versus their water controls (*t* (19) = 3.61, *p* = 0.002), while no alcohol-water difference was apparent in the females tested at this time (*t*-test, *p* = 0.57).

### 3.4. Porsolt Forced Swim Test

#### 3.4.1. Latency to Immobility/Floating

**Figure 9:**
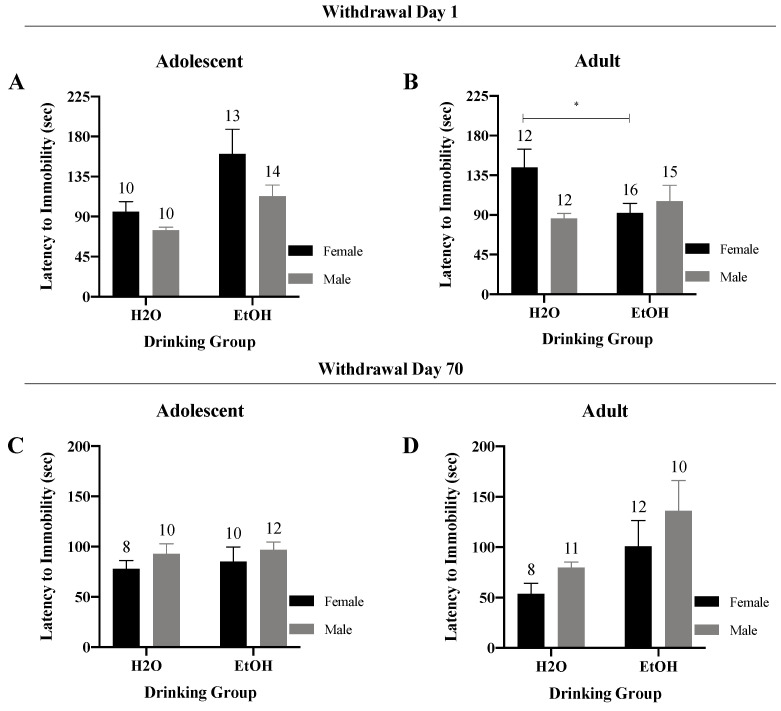
Summary of the results for the Sex × Drinking ANOVA observed for the latency to immobility in the Porsolt Forced Swim Test. (**A**) Depiction of the results for the adolescent-onset mice at 1-day withdrawal. (**B**) Depiction of the Sex × Drinking interaction detected for the female adult-onset mice at 1-day withdrawal. (**C**) Depiction of the results for the adolescent-onset mice at 70-days withdrawal. (**D**) Depiction of the results for the adult-onset mice at 70-days withdrawal. The data represent the means ± SEMs for the numbers of mice indicated in each panel. * *p* < 0.05 for female H2O vs. female EtOH.

In early withdrawal, a Sex × Drinking ANOVA conducted on the latency to first float by adolescent-onset mice indicated no significant interaction (Figure 9A) (*F*(1,43) = 0.54, *p* = 0.47); however, there was a significant main effect of Drinking Group (*F*(1,43) = 8.06, *p* = 0.01) that reflected a shorter latency to float in water- versus binge-drinking adolescents. Conversely, a significant Sex x Drinking interaction was detected for the adult-onset mice tested in early withdrawal (Figure 9B) (*F*(1,51) = 5.56, *p* = 0.02). Deconstruction of this interaction along the Sex factor revealed a shorter latency to float in binge-drinking versus water-drinking females (*t* (26) = 2.38, *p* = 0.03). In contrast, no significant alcohol effect was noted for males (*t*-test, *p* = 0.36).

In later withdrawal, no significant Sex × Drinking interaction was uncovered for either the adolescent-onset (Figure 9C) (*F*(1,36) = 0.02, *p* = 0.88) or adult-onset mice (Figure 9D) (*F*(1,37) = 0.04, *p* = 0.84). However, a significant main effect of Drinking was detected for the adult-onset mice, which reflected a longer latency to float in binge-drinking versus water controls (*F*(1,37) = 5.56, *p* = 0.02).

#### 3.4.2. Time Spent Immobile

**Figure 10:**
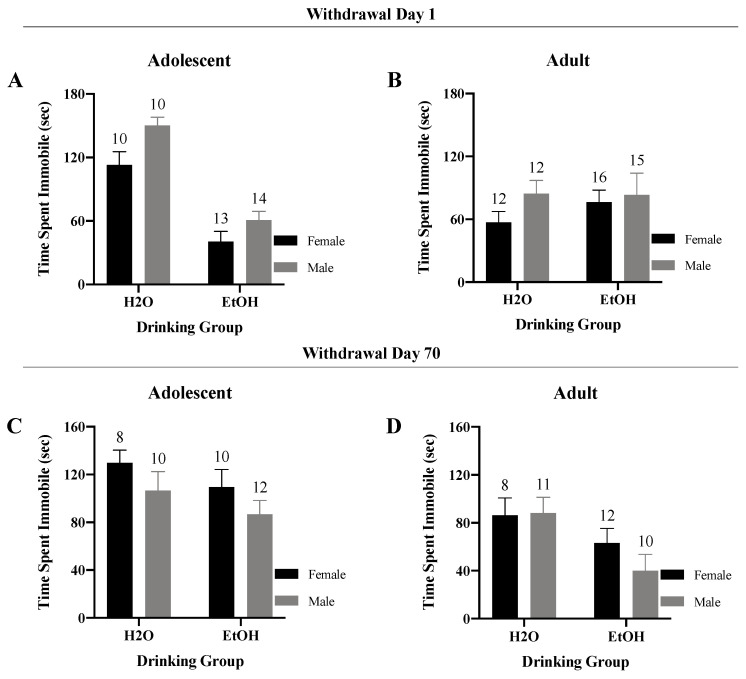
Summary of the results for the Sex × Drinking ANOVA observed for the total time spent immobile in the Porsolt Forced Swim Test. (**A**) Depiction of the results for the adolescent-onset mice at 1-day withdrawal. (**B**) Depiction of the results for the adult-onset mice at 1-day withdrawal. (**C**) Depiction of the results for the adolescent-onset mice at 70-days withdrawal. (**D**) Depiction of the results for the adult-onset mice at 70-days withdrawal. The data represent the means ± SEMs for the numbers of mice indicated in each panel.

Analyses of the time spent immobile during the forced swim test for the adolescent-onset mice tested in early withdrawal indicated no significant interaction (Figure 10A) (*F*(1, 43) = 0.77, *p* = 0.39), but significant main effects of both Sex (*F*(1,43) = 8.61, *p* = 0.01) and Drinking (*F*(1,43) = 68.36, *p* < 0.001). Overall, binge-drinking adolescents spent less time immobile versus water-drinking controls, with female mice spending less time immobile than their male counterparts. In contrast, no significant interactions (*F*(1,51) = 0.46, *p* = 0.50) or main effects (*p*’s > 0.05) were observed for the adult-onset mice tested in early withdrawal (Figure 10B).

In protracted withdrawal, the two-way ANOVA also failed to reveal a significant interaction for either the adolescent-onset (Figure 10C) (*F*(1,36) = 0.00, *p* = 1.00) or the adult-onset mice (Figure 10D) (*F*(1,37) = 0.86, *p* = 0.36). However, binge-drinking adult mice did spend significantly less time immobile than their water controls when tested in later withdrawal (Drinking effect: *F*(1,37) = 6.99, *p* = 0.01).

#### 3.4.3. Immobile Episodes

**Figure 11:**
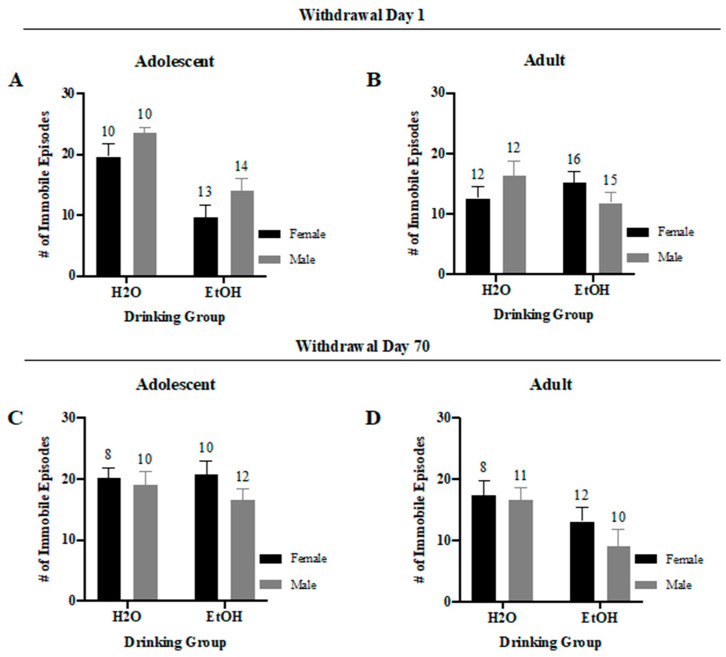
Summary of the results for the Sex × Drinking ANOVA observed for the total number of immobile episodes in the Porsolt Forced Swim Test. (**A**) Depiction of the results for the adolescent-onset mice at 1-day withdrawal. (**B**) Depiction of the results for the adult-onset mice at 1-day withdrawal. (**C**) Depiction of the results for the adolescent-onset mice at 70-days withdrawal. (**D**) Depiction of the results for the adult-onset mice at 70-days withdrawal. The data represent the means ± SEMs for the numbers of mice indicated in each panel.

In line with the data for the time spent immobile (Figure 10A), the data did not indicate a significant interaction for adolescent mice tested in early withdrawal (Figure 11A) (*F*(1,43) = 0.02, *p* = 0.89), but did show a significant main effect of both Sex (*F*(1,43) = 4.70, *p* = 0.04) and Drinking (*F*(1,43) = 25.35, *p* < 0.001), with binge-drinking mice exhibiting fewer immobile episodes than water-drinkers and female mice exhibiting less immobility than males. Interestingly, a notable trend for an interaction between Sex and Drinking Group was detected for the adult-onset mice tested in early withdrawal (Figure 11B) (*F*(1,51) = 3.34, *p* = 0.07). However, upon further analyses, no significant water-alcohol differences were observed for either female (*t*-test, *p* = 0.36) or male mice (*t*-test, *p* = 0.12).

For the animals in protracted withdrawal, the two-way ANOVA failed to determine a significant Sex × Drinking interaction for either adolescent-onset (Figure 11C) (*F*(1,36) = 0.57, *p* = 0.45) or adult-onset mice (Figure 11D) (*F*(1,37) = 0.49, *p* = 0.49). Nevertheless, a significant main effect of Drinking Group for the adult mice showed a higher number of immobile episodes for the water-drinking mice compared the binge-drinking adult mice in protracted withdrawal (*F*(1,37) = 6.28, *p* = 0.02).

### 3.5. Corticosterone Assay

#### 3.5.1. Basal Corticosterone

**Figure 12:**
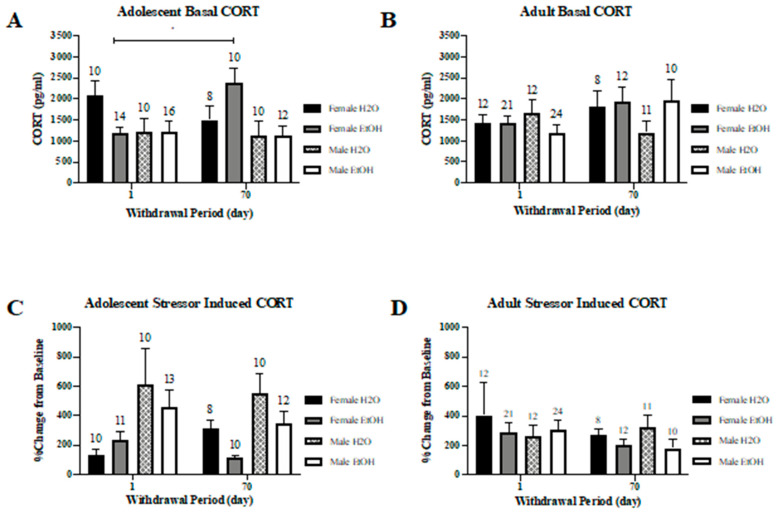
Summary of the subject factor interactions observed regarding plasma corticosterone (CORT). (**A**) Depiction of the Sex × Drinking × Withdrawal interaction observed for adolescent-onset drinking mice versus (**B**) the lack thereof for adult-onset drinking animals. (**C**) Depiction of the Sex × Drinking × Withdrawal interaction for stressor-induced corticosterone levels in adolescent-onset mice. (**D**) Depiction of the lack of group differences in stressor-induced corticosterone levels in adult-onset animals. The data represent the means ± SEMs for the numbers of mice indicated in each panel. * *p* < 0.05 WD1 vs. WD70 for female EtOH.

Analyses of the basal CORT levels of the adolescent-onset mice indicated a significant Sex × Drinking × Withdrawal interaction (*F*(1,82) = 4.77, *p* = 0.032). Thus, the interaction was deconstructed along the Withdrawal factor to examine for Sex × Drinking interactions at each withdrawal time-point. This deconstruction revealed no significant interaction for the adolescent mice tested on Withdrawal Day 1 (*p* = 0.096) or 70 (*p* = 0.170). The significant Sex × Drinking × Withdrawal interaction was then deconstructed along the Drinking factor to examine for withdrawal-dependent changes in basal CORT. In binge-drinking mice, a significant Sex × Withdrawal interaction was observed (*F*(1,48) = 6.86, *p* = 0.012), which reflected a withdrawal-dependent increase in basal CORT in the adolescent-onset female binge-drinkers (Figure 12A) (*t* (20) = 3.08, *p* = 0.006), but not in the other adolescent-onset groups (for female water, male water and male alcohol, *t*-tests, *p*’s > 0.05).

In contrast to the adolescent-onset mice, no group differences in basal CORT levels were detected in adult animals (Figure 12B) (*F*(1,102) = 1.79, *p* = 0.184).

#### 3.5.2. Stressor-Induced Corticosterone

A Sex × Withdrawal × Drinking ANOVA indicated no significant interactions for stressor-induced CORT levels in adolescent-onset mice (*F*(1,82) = 0.54, *p* = 0.47). However, a main effect of Sex was detected (*F*(1,82) = 65.33, *p* < 0.001), which reflected lower stressor-induced CORT levels in females versus males (Figure 12C). For the adult-onset animals (Figure 12D), the 3-way ANOVA yielded an insignificant interaction (*F*(1,102) = 0.703, *p* = 0.404) and resulted in no significant main effects (all *p*’s > 0.05).

#### 3.5.3. Inter-Relations between Alcohol Intake and Corticosterone Levels

When all mice were considered (*N* = 197), we did not find any significant correlations between the average alcohol intake of the mice and either basal CORT levels (*r* = 0.003, *p* = 0.974) or stressor-induced increases in CORT levels on the test day (*r* = −0.024, *p* = 0.79). As it might be predicted that alcohol consumption would have a greater impact upon basal and stressor-induced changes in CORT during early versus later withdrawal, we deconstructed the data along the Withdrawal factor for re-analysis. However, we still failed to detect significant relationships between alcohol intake and basal or stressor-induced changes in CORT, even at 1-day withdrawal (*r*’s < 0.51, *p*’s > 0.25; data not shown). Given the sex- and age-related differences in alcohol intake (Figure 2), we also deconstructed the data along these subject factors and conducted additional follow-up correlational analyses to determine whether or not sex- or age-related differences might exist for the inter-relationship between alcohol intake and our CORT measures. The only significant relationship that was detected was a positive one between alcohol intake and stressor-induced CORT in males (*r* (64) = 0.322, *p* = 0.001). The remainder of the results failed to indicate any significant relationships between alcohol consumption and CORT, even when only the data from withdrawal day 1 were considered (data not shown).

#### 3.5.4. Inter-Relations between Corticosterone Levels and Behavioral Indices of Negative Affect

**Figure 13:**
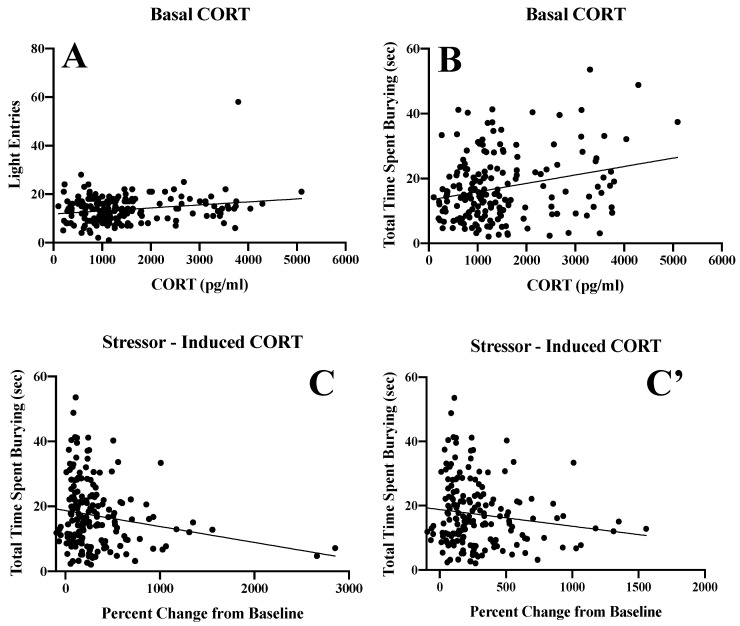
Summary of the significant inter-relations between corticosterone levels and behavioral indices of negative affect. A predictive relationship was observed between basal corticosterone (CORT) and (**A**) the number of light entries in the light–dark shuttle-box test and (**B**) the total time spent burying in the marble-burying test. (**C**) In contrast, when all animals were considered, an inverse relationship was observed between the stressor-induced changes in corticosterone and the time spent burying. (**C’**) Re-analysis of the data in Panel C following removal of the two outlier mice exhibiting very high stressor-induced CORT responses reduced the strength of this inverse relationship.

When all mice were considered, we did detect significant correlations between basal CORT levels and both the total number of light entries from the light dark box test (Figure 13A) (*r* (177) = 0.211, *p* = 0.005) and the total time spent marble-burying (Figure 13B) (*r* (177) = 0.25, *p* = 0.001). The total time spent marble-burying was also inversely correlated with stressor-induced CORT (Figure 13C) (*r* (177) = −0.18, *p* = 0.01). However, inspection of Figure 13C suggested that this correlation may be driven by two mice with very high stressor-induced CORT responses. Indeed, analysis indicated that the data for these two animals were two standard deviations above the mean of the population. Thus, the data from these two mice were removed and re-analysis revealed instead a strong statistical trend for a correlation (Figure 13C’) (r (175) = 0.14, *p* = 0.057). When all mice were considered, no other significant correlations were detected vis-à-vis the average total alcohol intake and our behavioral measures of negative affect (data not shown).

Although alcohol intake was found to be unrelated to either basal or stressor-induced CORT levels (see Section 3.5.3), we tested the possibility that the relationship between CORT and behavior might vary as a function of binge-drinking history by deconstructing the data along the Drinking factor prior to re-analysis. However, the results of this re-analysis failed to indicate any correlations that were specific to the alcohol-drinking mice (Table 1).

We also examined how our subject factors of Sex and Age might influence the relationship between basal and stressor-induced changes in CORT and our behavioral measures of negative affect. When all mice were considered, basal CORT levels predicted the time spent burying, a relationship that held only when females were included in the analysis (*r* (86) = 0.28, *p* = 0.01), although a strong positive trend was also observed when only males were considered (*r* (93) = 0.20, *p* = 0.055). The relationship also held up when only adults were examined (*r* (94) = 0.28, *p* = 0.007; for adolescents, *r* (85) = 0.20, *p* = 0.07), arguing that the positive correlation reflected primarily the results of the adult mice. When all mice were considered, stressor-induced CORT levels were inversely related to the time spent burying (Table 1). While this correlation did not hold upon deconstruction along the Sex and Age factors, it is noteworthy that the relationship between these variables trended strongly in female mice (*r* (86) = −0.20, *p* = 0.07; for males, *r*(91) = −0.15, *p* = 0.15) and adolescent mice (*r*(85) = −0.20, *p* = 0.07; for adults: *r*(94) = −0.14, *p* = 0.17).

### 3.6. Inter-Relations between Initial Alcohol Intake and Subsequent Alcohol Consumption

**Figure 14:**
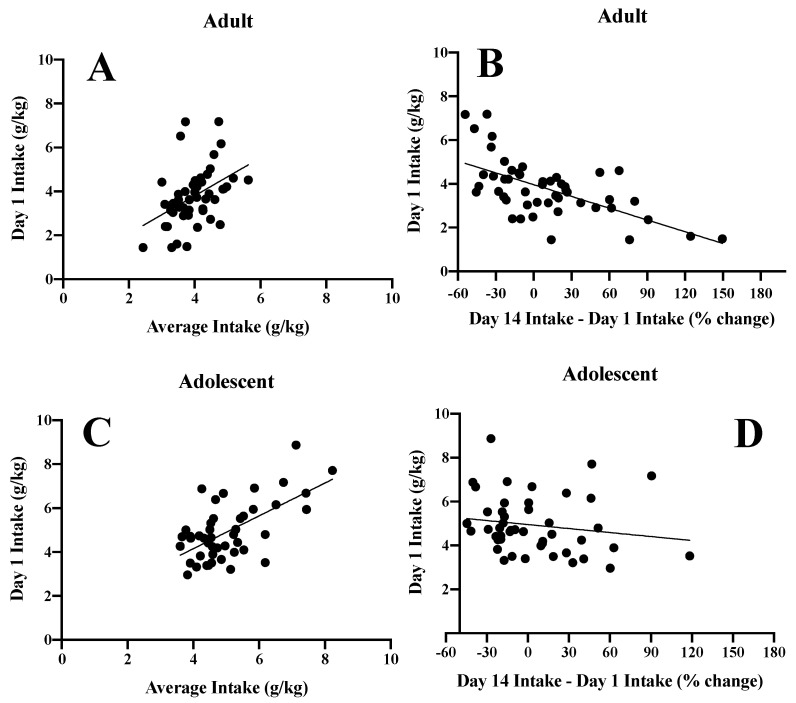
Summary of the significant inter-relations between measures of alcohol intake. A predictive relationship was observed between alcohol intake on Day 1 of drinking and the average alcohol intake over the 14-day drinking period in both (**A**) adult and (**C**) adolescent mice. Conversely, initial alcohol intake was inversely related to the escalation of intake observed over the course of the 14-day drinking period in both (**B**) adult and (**D**) adolescent mice.

As clear sex- and age-related differences existed with respect to alcohol intake (Figure 2), we conducted correlational analyses to determine whether or not group differences might exist with respect to the ability of initial alcohol intake to predict subsequent alcohol consumption. In adult mice, initial alcohol intake predicted their average total alcohol consumption (Figure 14A) (*r* (49) = 0.43, *p* = 0.002), but was inversely related to the extent to which alcohol intake escalated over the course of the 14-day drinking period (Figure 14B) (*r* (49) = −0.62, *p* < 0.0001). Initial alcohol intake also predicted the average alcohol consumption of adolescent mice (Figure 14C) (*r* (46) = 0.62, *p* < 0.0001), but did not reliably predict the extent of escalation (Figure 14D) (*r* (46) = −0.17, *p* = 0.27). Deconstructing the data along the Sex factor did not yield any significant, sex-specific, correlations that were distinct from those observed when both male and female subjects were combined (data not shown).

### 3.7. Inter-Relations between Indices of Alcohol Intake and Behavioral Indices of Negative Affect

Given the failure to detect many alcohol-induced changes in negative affect using omnibus ANOVA approaches, we conducted correlational analyses to determine whether or not binge-drinking history might predict the magnitude of negative affect manifested during alcohol withdrawal as an alternative statistical approach to our dataset. As the marble-burying test yielded results most consistent with our prior reports, in addition to a positive relationship between CORT levels and behavior (Table 1), we conducted correlational analyses between the behavioral measures from the marble-burying test and (1) the total alcohol consumption on the first day of binge-drinking, (2) the average total alcohol consumption across the 14 drinking days; and (3) the change in alcohol intake from Days 1 to 14 of alcohol-drinking (an index of drinking escalation). Curiously, when all binge-drinking mice were considered (*N* = 95–99), the average total alcohol intake was inversely correlated with the percent of marble buried (*r* = −0.25, *p* = 0.01), with no significant correlations detected regarding the relationship between initial alcohol intake and the escalation of alcohol intake and our three behavioral measures in this assay (*r*’s < 0.149, *p*’s > 0.20; data not shown).

**Figure 15:**
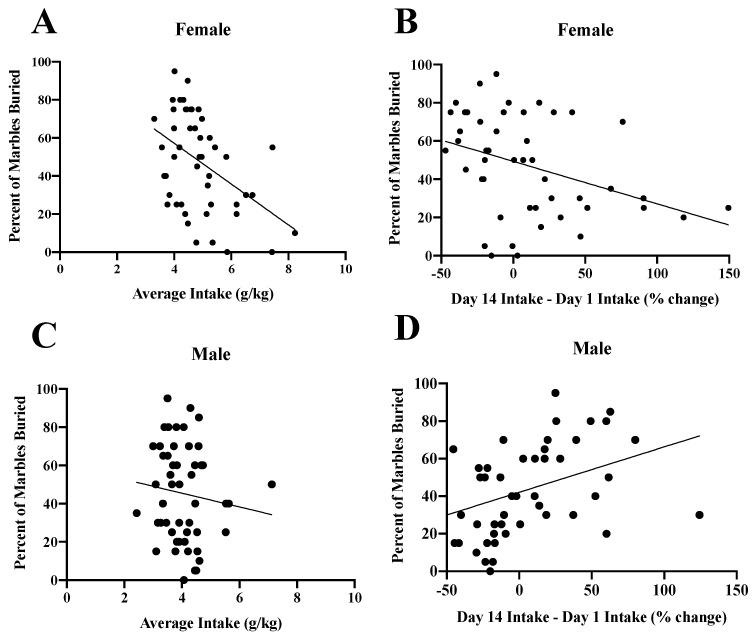
Summary of the inter-relations between the average alcohol intake (left), the escalation of alcohol intake (right) and the percent marble buried in the marble-burying test. In female mice, an inverse relationship was observed between both (**A**) alcohol intake and (**B**) the escalation of alcohol intake and the percent of marbles buried. (**C**) In male mice, no significant correlation was observed between the average alcohol intake and the percent of marbles buried. (**D**) However, an escalation of drinking predicted the percent of marbles buried in males.

Given the age- and sex- related differences in alcohol intake, the data were the deconstructed along these factors for re-analysis of their influence upon the relationship between our drinking measures and anxiety-like behavior in the marble-burying test. Consistent with the data from all mice mentioned above, initial alcohol intake did not predict any aspect of marble-burying behavior when the data was examined as a function of sex, age of drinking onset or withdrawal (data not shown). Curiously, when all female mice were considered, both the average alcohol intake, as well as the escalation of alcohol intake over the 14-day drinking period, predicted lower signs of anxiety-like behavior in the marble-burying test, as indicated by a positive relationship between the drinking measures and the latency to bury (Table 2) and an inverse relationship between the drinking measures and both the time spent burying (Table 2) and the percent of marble buried (Figure 15A,B; Table 2). Although the average alcohol intake did not predict subsequent marble-burying behavior in male mice (% buried in Figure 15C; see also Table 2), the extent to which alcohol intake escalated during the binge-drinking phase of the study was positively correlated with both the time spent burying and the percentage of marbles buried (Figure 15D; Table 2). In contrast to the outcomes of the sex-related analysis, we failed to detect any major influence of the Age factor upon the relationships between alcohol intake and behavior in the marble-burying test (Table 2).

## 4. Discussion

The goal of the present study was to directly interrogate sex differences in the age-related effects of binge-drinking upon negative affect expressed during early and protracted (70 days) withdrawal. We expected to replicate our prior observations from male mice indicating an interaction between the age of drinking-onset and withdrawal upon negative affect [10,24,25,26,41,42,43] and more recent findings suggesting a persistent increase in withdrawal-induced negative affect in female animals [35]. Based on evidence that female rodents tend to consume more alcohol than males [28,29,30,31,32,33,34,35,36,37,38], we hypothesized that female binge-drinking animals would exhibit more pronounced and/or enduring signs of withdrawal-induced negative affect than their male counterparts, irrespective of the age of drinking-onset. Using a 4-bottle-choice DID procedure, we replicated both age- [35,36,37] and sex-related [28,29,30,31,32,33,34,35,36,37,38] differences in alcohol binge-drinking in mice, with adolescents consuming more alcohol than adults and females consuming more alcohol than males. The BACs for the adult-onset binge-drinking males were quite variable and their mean BAC on the day of sampling was just shy of the NIAAA 80 mg/dL criterion for binge-drinking [1]. However, alcohol intake resulted in BACs ≥ 80 mg/dL for the other groups tested, which is a finding in line with our prior studies using a 3-bottle-choice (10, 20, and 40% alcohol) DID drinking procedure [25,26,35,41,42]. Despite the sex difference in alcohol intake, we detected very few sex differences in the manifestation of withdrawal-induced negative affect. More concerning, when both sexes were tested concurrently, we failed to replicate not only age-dependent differences in withdrawal-induced negative affect in male mice but the direction of our alcohol effects tended to be opposite those reported in our prior studies of a single sex [10,24,25,26,35,41,42,43].

Adult, male, mice with a 2-week history of binge-drinking under 3- or 4-bottle-choice DID procedures exhibit robust signs of anxiety-like behavior at one day withdrawal that dissipates by 30 days withdrawal [10,24,25,26,41,42,43]. In contrast, male mice with a 2-week history of binge-drinking during adolescence are “resilient” to the negative affective state produced early in alcohol withdrawal, but a negative affective state incubates during alcohol withdrawal, manifesting robustly when the mice are adults [10,24,25,26,42]. In our laboratory, this interaction between the age of binge-drinking-onset and alcohol withdrawal can be reliably detected in males, when mice are tested under light–dark shuttle-box, marble-burying, and forced swim procedures; other tests of anxiety-like behavior, such as the novel object encounter and elevated plus-maze, are less sensitive to the effects of alcohol withdrawal upon anxiety-like behavior, even for mice with a more extensive, 30-day, binge-drinking history [43]. While the specific variables demonstrating alcohol-water differences in negative affect do vary from report to report, the direction of the alcohol-water differences reported for both male [10,24,25,26,41,42,43] and female [35] mice have been consistent with the interpretation that alcohol withdrawal induces a negative affective state. Moreover, in our hands, the light–dark shuttle-box, marble-burying and forced swim tests have also proven to be sensitive to age-related differences in basal anxiety-like behavior in alcohol-naïve mice, with the behavior of alcohol-naïve adolescents aligning with the interpretation of a hyper-anxious phenotype, particularly for males [10,24,25,26,41,42,43,50]. The reliability of our findings over the past several years is precisely the reason for selecting these behavioral paradigms for this large-scale study of the role for biological sex in mediating alcohol withdrawal-induced negative affect.

Indeed, some of the results of the present sex difference study do corroborate our earlier findings from studies employing a single sex. Male, adult-onset binge-drinking mice exhibited a longer latency to enter the light-side of shuttle-box on withdrawal Day 1—an effect no longer apparent at 70 days withdrawal (Figure 3B). In the marble-burying assay, female adolescent-onset, binge-drinking mice exhibited a shorter latency to begin marble-burying at one day withdrawal and this effect persisted for at least 70 days (Figure 4A). In contrast, male adolescent-onset, binge-drinking mice exhibited a shorter latency to begin burying only at the 70-day withdrawal time-point (Figure 4A)—a finding consistent with an incubation of a negative affective state in male adolescent drinkers [24] and the first demonstration by our group that incubated affective state persists beyond 30 days withdrawal. Additionally, both male and female adult-onset, binge-drinking mice exhibited a shorter latency to begin marble-burying on withdrawal day 1, relative to their water-drinking controls (Figure 4B). However, in contrast to prior reports [10], this effect was still apparent at 70 days withdrawal (Figure 4B). Although no significant sex difference was detected by omnibus ANOVA, the enduring nature of the effect of adult-onset binge-drinking upon the latency to marble-bury aligns with that reported previously for binge-drinking, female mice [35] and may be driven largely by the female subjects.

To the best of our knowledge, only one other published study has attempted to examine directly for sex differences in interactions between the age of binge-drinking onset under DID procedures, alcohol withdrawal and negative affect [35]. In this earlier study from our group, all of the male and female mice exhibited BACs considerably lower than typically observed under DID procedures (~30 to 70 mg/dL)—a finding we eventually attributed to an insufficient period of acclimation to the drinking cages prior to alcohol presentation [35]. Not surprisingly given the low BACs of the mice, we detected no water–alcohol differences in negative affect in this earlier study, precluding any conclusions regarding sex differences or subject factor interactions in our affective measures [35]. In contrast to our earlier report [35], the BACs detected herein were near to, or above, the 80 mg/dL NIAAA criterion for binge-drinking (Figure 2F). Despite this, the vast majority of the affective measures in the present study either failed to indicate water-alcohol differences (e.g., marble-burying; Figure 4) or indicated a counter-intuitive result whereby water controls exhibited more anxiety-like behavior than their alcohol-experienced counterparts. While the interpretation of the direction of alcohol’s effects upon behavior in the forced swim test is controversial (see Ref. [41] for discussion), we were particularly struck by relatively high levels of anxiety-like behavior exhibited by water controls in the light–dark shuttle-box test and the polar opposite water-alcohol differences observed for both adult and adolescent mice in this paradigm (Figure 3) versus those reported by our group previously in studies of either male [24,25,26,42,43] or female [35] mice. While it is true that marked procedural differences existed with respect to the daily handling and housing of water- versus alcohol-drinking animals in the present study, the procedures employed herein where nearly identical to those employed in our published work over the past 3 years in which very clear alcohol-water differences in affective behavior were detected in both male [24,25,26,42] and female mice [35]. In fact, the first several cohorts of this sex difference study were conducted in parallel with some of the later “female only” cohorts summarized in Szumlinski et al. (2019), which successfully replicated many of our reported effects of alcohol withdrawal upon affective behavior in female subjects [35].

We were also struck by the very few age-related differences in basal anxiety-like behavior exhibited by water-drinking controls during early withdrawal in the present study as our prior work reliably detected higher behavioral indices of anxiety in adolescent versus adult males [10,24,25,26,41,42,43] and females [35]. Herein, water-drinking adolescents exhibited the shortest latency to begin floating (Figure 9), as well as most floating behavior (Figure 10 and Figure 11), when assayed on WD1—findings indicative of age-related differences in the basal affective response to, or coping strategy employed in, the forced swim test. Curiously, this is the first time we have detected adolescent-adult differences in the floating behavior manifested by water-drinking controls on WD1; in our earlier reports, the amount of floating/swimming, as well as the latency to first float, were both comparable between water-drinking adolescent and adult mice tested on WD1, although marked differences in anxiety-like behavior were detected in the light–dark box and marble-burying tests [10,35]. Aligning with our published studies in mice [10,24,25,26,35,41,42,43], age-related differences in affective measures have been consistently reported in drug/alcohol-naïve rats, as has a resiliency to the negative affective state produced by early alcohol withdrawal in adolescent animals [5,7,9]. Thus, both the relative lack of adolescent-adult differences in baseline emotionality (particularly in the light–dark box and marble-burying tests) and in the responsiveness to early alcohol withdrawal were very unexpected. At the present time, it is unclear why our adolescent animals behaved so differently from the mice in our prior work. This being said, one major procedural difference between this and prior work (at least from our laboratory) relates to the concurrent testing of male and female subjects. In partial support of this, chemosensory social stimuli, such as those in vaginal secretions, differentially alter neuronal activity within the mesocorticolimbic system of adolescent versus adult males to affect motivated behavior [51,52,53]—an effect attributed to the differential maturation state of the brain, rather than changes in circulating testosterone [52,53]. While this line of chemosensory research has focused on measures of conditioned reward, it is entirely possible that exposure to pheromones from adults of the opposite sex during anxiety testing may have unpredictably impacted the behavior of the adolescent mice in the present study.

Related to this, concurrent testing of male and female subjects may have also mitigated the negative affective state produced by a history of binge-drinking in adults. In support of this notion, exposure to adult female urinary pheromones during elevated plus-maze testing produces a testosterone-driven anxiolytic effect, without impacting locomotor activity in this assay [54]. Such a finding aligns with other research indicating an anxiolytic effect of circulating testosterone in adult male laboratory mice and rats, to include behavior in the marble-burying test [55,56]. Thus, the blunted negative affective state produced by alcohol withdrawal exhibited by the male mice herein could very well reflect a testosterone-dependent anxiolysis, elicited by the presence of female pheromones during testing. While it is known that emotionality varies with the estrous cycle in adult female rodents [57], to the best of our knowledge, it remains to be determined whether exposure to adult male pheromones elicits a comparable anxiolytic effect in either adolescent or adult female subjects to account for their blunted negative affective state observed herein. Future work seeks to better understand how exposure to urinary pheromones from the opposite sex impacts anxiety-related measures in both adult and adolescent mice to alter the expression of such measures during alcohol withdrawal.

## Figures and Tables

**Table 1:** Summary of the inter-relations between basal and stressor-induced increases in corticosterone (CORT) and our behavioral measures of negative affect, deconstructed along the between-subjects factor of Drinking. Sample sizes are indicated in parentheses. Significant correlations are indicated in bold.

Drinking History	Dependent Variable	Basal CORT	Stressor-Induced CORT
Water (*N* = 81)	Latency to Bury	*r* = 0.155, *p* = 0.168	*r* = −0.137, *p* = 0.223
	Time in light	*r* = 0.065, *p* = 0.562	*r* = 0.041, *p* = 0.717
	Light Entries	***r* = 0.229, *p* = 0.039**	*r* = 0.011, *p* = 0.921
	Latency to Bury	*r* = 0.155, *p* = 0.168	*r* = −0.137, *p* = 0.223
	Time Spent Burying	*r* = 0.068, *p* = 0.547	*r* = −0.111, *p* = 0.325
	Percent Buried	*r* = −0.069, *p* = 0.541	*r* = 0.077, *p* = 0.493
	Latency to Immobility	*r* = 0.049, *p* = 0.667	*r* = −0.082, *p* = 0.466
	Time Spent Immobile	*r* = −0.042, *p* = 0.711	*r* = 0.067, *p* = 0.552
	Immobile Episodes	*r* = −0.092, *p* = 0.412	*r* = 0.076, *p* = 0.501
Alcohol (*N* = 98)	Latency to enter light	*r* = −0.107, *p* = 0.296	*r* = 0.025, *p* = 0.807
	Time in light	*r* = −0.133, *p* = 0.193	*r* = 0.116, *p* = 0.254
	Light Entries	***r* = 0.203, *p* = 0.045**	*r* = 0.018, *p* = 0.860
	Latency to Bury	***r* = −0.216, *p* = 0.033**	***r* =** **0.354** **, *p* < 0.001**
	Time Spent Burying	***r* = 0.397, *p*< 0.001**	***r* = −0.262** **, *p* = 0.009**
	Percent Buried	***r* = 0.216, *p* = 0.033**	*r* = −0.171, *p* = 0.093
	Latency to Immobility	*r* = −0.005, *p* = 0.962	*r* = −0.003, *p* = 0.974
	Time Spent Immobile	*r* = 0.031, *p* = 0.763	*r* = 0.077, *p* = 0.448
	Immobile Episodes	*r* = 0.084, *p* = 0.410	*r* = 0.059, *p* = 0.561

**Table 2:** Summary of the inter-relations between the average alcohol intake and measures of anxiety-like behavior in the marble-burying test, expressed as a function of our between-subjects factors. the average alcohol intake and basal, as well as stressor-induced increases in, corticosterone (CORT) as a function of the independent variables investigated. The number of mice included in each individual analysis is indicated in parentheses. Significant correlations are indicated in bold. WD = withdrawal day.

Subject Factor	Measure	Latency to Bury	Time Burying	% Buried
Females (*N* = 49)	Ave. Intake	***r* = 0.313, *p* = 0.028**	*r* = −0.204, *p* = 0.160	***r* = −0.442, *p* = 0.001**
	Escalation	***r* = 0.327, *p* = 0.023**	***r* = −0.309, *p* = 0.033**	***r* = −0.373, *p* = 0.009**
Males (*N* = 50)	Ave. Intake	*r* = 0.224, *p* = 0.119	*r* = −0.146, *p* = 0.313	*r* = −0.113, *p* = 0.435
	Escalation	*r* = −0.136, *p* = 0.361	***r* = 0.298, *p* = 0.042**	***r* = 0.372, *p* = 0.010**
Adolescents (*N* = 46)	Ave. Intake	*r* = −0.117, *p* = 0.439	*r* = −0.141, *p* = 0.350	*r* = −0.145, *p* = 0.335
	Escalation	*r* = −0.123, *p* = 0.415	*r* = 0.135, *p* = 0.371	*r* = 0.150, *p* = 0.319
Adults (*N* = 53)	Ave. Intake	*r* = 0.260, *p* = 0.060	*r* = −0.023, *p* = 0.873	*r* = 0.015, *p* = 0.915
	Escalation	*r* = 0.271, *p* = 0.060	*r* = −0.106, *p* = 0.272	*r* = −0.215, *p* = 0.139
